# Microgreens Production with Low Potassium Content for Patients with Impaired Kidney Function

**DOI:** 10.3390/nu10060675

**Published:** 2018-05-26

**Authors:** Massimiliano Renna, Maria Castellino, Beniamino Leoni, Vito Michele Paradiso, Pietro Santamaria

**Affiliations:** 1 Department of Agricultural and Environmental Science, University of Bari Aldo Moro, Via Amendola 165/A, 70126 Bari, Italy; beniamino.leoni@uniba.it (B.L.); pietro.santamaria@uniba.it (P.S.); 2 Department of Soil, Plant and Food Science, University of Bari Aldo Moro, Via Amendola 165/A, 70126 Bari, Italy; maria.castellino@uniba.it (M.C.); vitomichele.paradiso@uniba.it (V.M.P.)

**Keywords:** antioxidant activity, *Cichorium intybus* L., hydroponic system, *Lactuca sativa* L., potassium intake, proximate composition

## Abstract

Chronic kidney disease represents a global problem together with other so-called ‘lifestyle-related diseases’. Unlike the healthy population, for the patients with impaired kidney function, it is of course prudent to recommend a restriction of high-potassium foods. Thus, it is suggested to limit the consumption of vegetables, because they generally contain high concentrations of potassium. At the same time, a lower consumption of vegetables reduces the intake of healthy compounds such as vitamins, fibers, and antioxidants, which also reduces the vegetables’ potential benefit in chronic kidney disease patients. Microgreens are an emerging class of specialty crop that represent a nutritious and refined food. In this study, for the first time, some chicory (local variety ‘Molfetta’ and cultivar ‘Italico a costa rossa’) and lettuce (cultivar ‘Bionda da taglio’) genotypes were grown using a hydroponic system with different potassium (K) levels (0, 29.1, 58.4, and 117 mg L^−1^) in order to produce microgreens with a low potassium content. The crop performances, cations content, proximate composition, and antioxidant activity were analyzed. Independent of the genotype, the K content in the microgreens was successfully reduced using a nutrient solution (NS), without K or with 29.1 mg K L^−1^, which supplied between 103 and 129 mg of K 100 g^−1^ FW (about 7.7–8.6% of the K daily intake that was recommended for the patients that were affected by chronic kidney disease). Whereas, 100 g of microgreens that were grown by using an NS with 58.4 or 117 mg K L^−1^ supply between 225 and 250 mg of K (about 15.8–16.5% of the K daily intake recommended for patients affected by chronic kidney disease). No differences were observed in terms of the shoot height, dry matter, proximate composition, and visual quality. A slightly lower yield was observed using an NS with a K concentration <58.4 mg L^−1^. These results suggest that by using an NS without K or with low K concentrations, it is possible to obtain a useful reduction of K in microgreens, without negatively affecting the quality. Unlike conventional vegetables, the microgreens that were produced in the present study could reduce the potassium intake in patients with impaired kidney function who were accustomed to eating vegetable-based dishes.

## 1. Introduction

For the human body, potassium (K) is an essential nutrient that is involved in fluid, acid, and electrolyte balance and it is required for normal cellular function, such as DNA and protein synthesis [1]. It is well known that K can modulate blood pressure, and that an increasing dietary K intake is associated with lower blood pressure. For this reason, the World Health Organization [2] recommends a K intake of at least 3510 mg day^−1^, to decrease the risk of cardiovascular diseases. Based on the estimates of the current K intakes in Europe (up to 5–6 g day^−1^ in adults), the risk of adverse effects from the K intake from food is considered to be low for the generally healthy population [3]. Nevertheless, certain groups, particularly those with impaired an kidney excretion of potassium, are sensitive to the adverse effects of increasing the K intake on heart function that is associated with increases in the plasma potassium. These groups firstly include subjects with chronic kidney disease, one of the so-called ‘lifestyle-related diseases’, together with hyperlipidemia, hypertension, and diabetes [4]. Chronic kidney disease represents a global problem, and it is estimated that by 2020, the number of dialysis patients will be around 3.8 million [5]. Unlike the healthy population, the K intake for the patients that are affected by chronic kidney disease is often restricted to 1500 mg day^−1^ [6]. In addition to these subjects, patients that also use pharmaceuticals that affect the kidney excretion of potassium, such as mineralocorticoid receptor antagonists, should be considered. Therefore, for all of these patients, the K intake must be restricted in order to avoid adverse effects on heart function as a result of disturbances in the plasma potassium concentration, most commonly known as hyperkalemia [7]. It is important to highlight that some investigators have begun to question the need to severely restrict potassium in chronic kidney disease patients, given the relative lack of published studies that support this strategy. However, in the absence of empirical evidence and conclusive data, it is of course prudent to continue to recommend a restriction of high-potassium foods for patients with impaired kidney function [8].

Since vegetables contain high concentrations of potassium [9,10], it is suggested that the patients with impaired kidney function do not take raw vegetables, rather, they should be soaked in water and boiled in order to reduce the K content through leaching [11]. Nevertheless, a K reduction by these cooking methods can be considered limited, while other important minerals and hydrophilic vitamins could be significantly lost [12,13,14]. In addition, it should be considered that people are also accustomed to eating raw vegetables, which makes it difficult to propose some vegetable-based dishes for patients with impaired kidney function. For example, eating raw salads is desirable for the dialysis patients and all of the subjects with an impaired renal excretion of potassium. Therefore, a dietary intake of vegetables that contains a lower K than usual could be a very useful health prevention method for the patients with impaired kidney function, which limits a decrease in the quality of life. In this context, the production of vegetables with low K content could be of great interest for research, considering that limited information is being reported in the literature [15,16,17]. 

Microgreens are an emerging class of specialty crop that can be considered as young and tender edible seedlings that are produced from the seeds of vegetables, herbs, or grains, including wild species. Depending on the species and growing conditions, microgreens are generally harvested 7–21 days after germination, when the cotyledon leaves have fully developed, and the first true leaves have emerged [18]. Microgreens can be used to enhance salads or as edible garnishes to embellish a wide variety of other dishes. Moreover, Renna et al. [19] has developed a new culinary concept that consists of considering self-produced microgreens as basic ingredients for the preparation of sweet and savory dishes, with interesting quality traits. At the same time, it has been reported that microgreens can provide higher amounts of phytonutrients (ascorbic acid, β-carotene, α-tocopherol, and phylloquinone) and minerals (Ca, Mg, Fe, Mn, Zn, Se, and Mo), compared with their mature-leaf counterparts [18]. Thanks to their distinctive peculiarities, microgreens represent a rich food source for particularly demanding categories of consumers, like vegetarians and vegans, who can diversify and enrich their diet using a large variety of microgreens that are available. Moreover, as the microgreens are usually consumed raw, they can also satisfy the specific needs of the so-called ‘raw foodists’ [20]. Lastly, the chance of growing microgreens in a very simple way, even in very little spaces, means that microgreens garner immense potential for adapting leafy vegetable production to a micro-scale and for improving nutritional value in the human diet [18,21]. This allows microgreens to be considered as ideal candidates for astronaut’s functional salads and, therefore, as a component of space-life support systems [22]. 

To the best of our knowledge, the literature absolutely lacks information on microgreens production, without or with a low potassium content. Therefore, starting from the above-mentioned considerations, the aims of the present study are as follows: (i) to investigate on the impact of reduced potassium concentrations in nutrient solution on the K content of microgreens and (ii) to assess the yield and nutritional quality of the microgreens that are grown using nutrient solutions with different potassium concentrations.

## 2. Materials and Methods

### 2.1. Experimental Conditions

Two experiments were conducted using a hydroponic system during the spring of 2017 in a green-house located at the University of Bari Aldo Moro (41°06′ N, 16°52′ E; Southern Italy). The first experiment was carried out from 5 to 18 April, while the second experiment was carried out from 20 April to 3 May. The experimental factors were (i) three genotypes (G) and (ii) three levels of K in the nutrient solution (NS). 

Two different G of chicory (*Cichorium intybus* L.) and one G of lettuce (*Lactuca sativa* L. Group *crispa*) were grown. The chicory seeds of the local variety ‘Molfetta’ (CM—a stem chicory type) and cultivar ‘Italico a costa rossa’ (CI—a leafy chicory type) were used, while a cultivar ‘Bionda da taglio’ (LB) was used for the lettuce seeds. All of the seeds were purchased from the Riccardo Larosa Company (Andria, Italy). The seeds were of a high quality, with a 95% germination at a constant temperature of 20 °C. A hydroponic tray system for the microgreens culture was created using polyethylene terephthalate fiber pads (50 cm × 24 cm × 0.89 cm; Sure to Grow^®^; Sure to Grow, Beachwood, OH, USA) as a growing medium that was placed on a 2.7 m^2^ (1 × 2.7 m) aluminum bench. The seeds were uniformly broadcasted on the surface of the growing media using a seeding density of four seeds cm^−2^. The sown fiber pads were irrigated manually using a water-nozzle and were covered with a black polyethylene film until the germination was complete.

The seedlings were fertigated by subirrigation with NS, having three levels of K. For the first experiment, the K levels were 0, 58.4 and 117.0 mg L^−1^, while for the second experiment, the K levels were 0, 29.1 and 58.4 mg L^−1^. A split-plot design with three replications was used. In all of the cases, the NS were prepared with pre-collected raining water containing (mg L^−1^) the following: 119 nitrogen, 16 phosphorus, 24 magnesium, 116 calcium, 54 sulfur, 1.12 iron, 0.27 manganese, 0.13 zinc, 0.27 boron, 0.03 copper, and 0.01 molybdenum, which resulted in an EC of 1.8 dS m^−1^ and pH 6.3. The NO_3_-N:NH_4_-N at a percentage ratio of 84:16 was used for the nitrogen source. The NS was delivered on the bench by a drip tape line with pressure-compensated drippers (each with a delivery rate of 7.0 L h^−1^). The drainage was collected in a reservoir tank at the base of the bench, but it was not reused (open cycle management).

**Figure 1:**
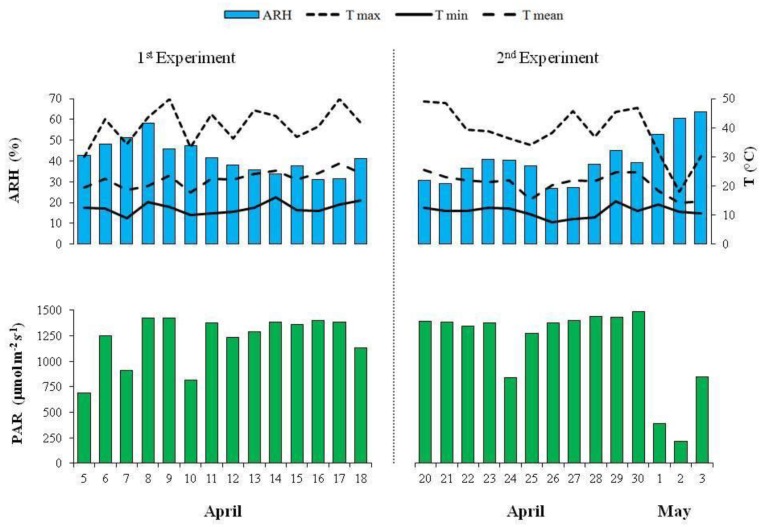
Average relative humidity (ARH), average mean, minimum (min) and maximum (max) air temperatures (broken lines), and photosynthetically active radiation (PAR) inside the greenhouse over the first and second experiment.

The values of the average relative humidity, average mean, minimum and maximum air temperatures, and photosynthetically active radiation inside the greenhouse, over the first and second experiment, are reported in Figure 1.

### 2.2. Harvesting, Yield Assessment, and Samples Preparation

**Figure 2:**
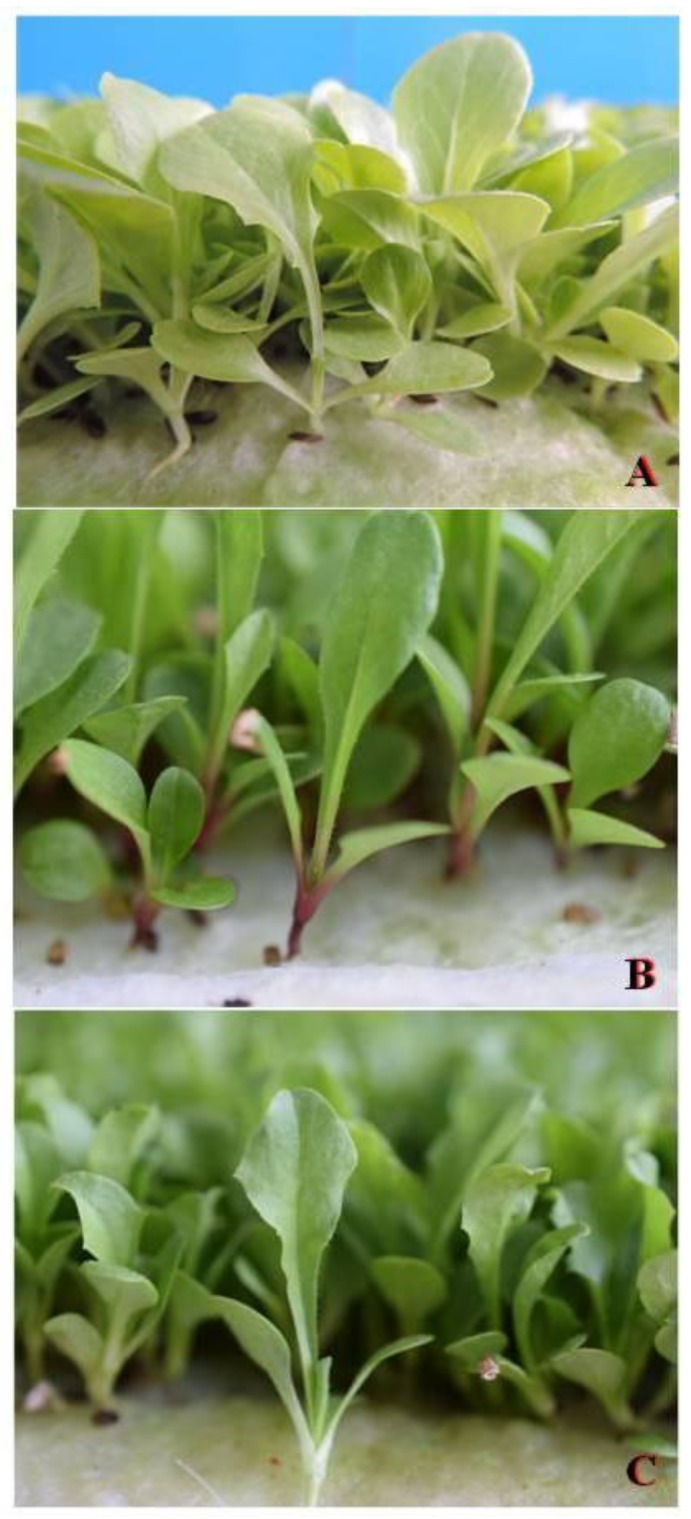
Harvesting stage (first true-leaves) of microgreens grown with 0 mg K L^−1^ in the nutrient solution, namely: (**A**) lettuce cultivar ‘Bionda da taglio’; (**B**) chicory cultivar ‘Italico a costa rossa’; and (**C**) chicory local variety ‘Molfetta’.

At the first appearance of the first true-leaves (Figure 2), the shoot height was measured, after which the microgreens were harvested by cutting the seedling just above the surface of the growing media with a knife. The harvested microgreens were weighed to determine the shoot fresh weight (FW) per unit area. The sub-samples were dried to a constant weight in a forced-draught oven at 65 °C and were weighed so as to determine their dry matter (DM) concentration. The oven dried samples were used for cation contents determination. The other sub-samples were freeze-dried (ScanVac CoolSafe 55-9 Pro; LaboGene ApS, Lynge, Denmark) and then used for proximate analysis and antioxidant activity determination.

### 2.3. Chemical Analysis

The proximate analysis of the samples was carried out as follows: ashes were determined by muffle furnace according to AOAC method 923.03 [23]; proteins content (N × 6.25) was determined by Kjeldahl nitrogen, according to the AOAC method 955.04 [23]; fat content was determined by Soxhlet extraction, according to the AOAC method 920.39 [23]; dietary fiber content was determined by the enzymatic-gravimetric procedure, according to the AOAC method 991.43 [23]; and total carbohydrates were calculated by the difference of protein, lipid, and ash on the dry matter basis.

The effect of different K supplementations on the antioxidant activity of microgreens by electron transfer mechanisms was evaluated using the 2,2-diphenyl-1-picrylhydrazyl (DPPH) stable radical scavenging capacity test, according to Difonzo et al. [24]. The freeze dried samples (0.1 g) were extracted with 5 mL methanol:water (80:20) for two hours, in tubes that were covered with aluminum foil. The extracts were then centrifuged for 15 min at 15,000 g and 24 °C. The supernatant was recovered and filtered with polytetrafluoroethylene (PTFE) septa (0.45 μm). The extracts (50 μL) were added to 950 μL of 0.08 mM DPPH in methanol. The mixture was shaken and left at room temperature in the dark for 30 min. The decrease of the absorbance at 517 nm was measured using a Cary 60 Agilent spectrophotometer (Agilent Technologies, Milan, Italy). The results were expressed in μmol Trolox equivalents (TE) 100 g^−1^ FW. Each sample was analyzed in triplicate.

For the inorganic ion content, an ion exchange chromatography (Dionex DX120; Dionex Corporation, Sunnyvale, CA, USA) with a conductivity detector was performed, as reported by D’Imperio et al. [25]. The cation contents (Na^+^, K^+^, Mg^2+^, and Ca^2+^) were determined in 1 g of dried sample, using an IonPac CG12A guard column and an IonPac CS12A analytical column (Dionex Corporation).

### 2.4. Statistical Analysis

The data were analyzed by a two-way analysis of variance (ANOVA), using the general linear model procedure of SAS software (SAS Version 9.1, SAS Institute, Cary, NC, USA). All of the means were compared using Student-Newman–Keuls (SNK) test at *p* = 0.05.

## 3. Results

### 3.1. Crop Performance

**Figure 3:**
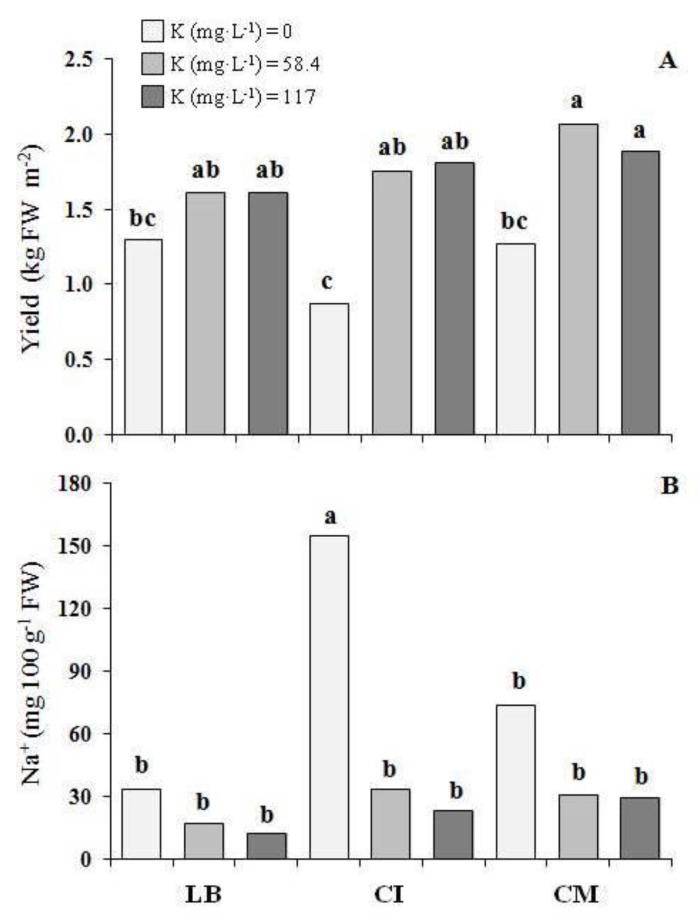
(**A**) Yield and (**B**) Na^+^ content of three genotype of microgreens grown with three potassium levels (first experiment). LB—lettuce ‘Bionda da taglio’; CI—chicory ‘Italico a foglia rossa’; CM—chicory ‘Molfetta’. For each histogram, the same lowercase letters indicate that the mean values are not significantly different (*p* = 0.05).

In the first experiment, the CM that was grown with 58.4 and 117 mg K L^−1^ showed the highest yield, about 72% higher on average than that of all of the genotypes that were grown with 0 mg K L^−1^ (Figure 3A). 

At the same time, the K level in the NS did not affect the yield for LB, while for the CI that was grown with 58.4 and 117 mg K L^−1^, the yield increased by 105% with respect to the 0 K level (Figure 3A). No differences were observed in terms of the shoot height (4.1 cm, on average) and dry matter (7.05 g 100 g^−1^ FW, on average) (Table 1).

In the second experiment, the highest yield was observed for the CM followed by the CI and LB, while 58.4 mg K L^−1^ obtained a yield 22% higher, compared with the other levels of K in the NS (Table 2). With regards to the shoot height, the CM and CI showed the highest values, while no differences were observed in terms of the dry matter (5.79 g 100 g^−1^ FW, on average).

### 3.2. Cation Contents

In the first experiment, the highest sodium content was found in the CI that was grown with 0 mg K L^−1^ (Figure 3B and Table 1). No differences in the potassium content were found between the three G, while with 0 level of K in the NS the potassium content in microgreens was 46% lower compared with 58.4 and 117 mg K L^−1^ (Table 1). Also for magnesium no differences were found between the three G, while 58.4 and 117 mg K L^−1^ decreased the magnesium content in the microgreens by 22% and 47%, respectively, compared with the 0 level of K in the NS. With regards to the calcium, the CI showed the highest content in the microgreens, while with 0 level of K in the NS, the calcium content in the microgreens was 50% higher, compared with ones grown with 117 mg K L^−1^ (Table 1).

In the second experiment, the K level in the NS did not affect the sodium content in the microgreens, while the LB showed a content that was 57% lower compared with the CI and CM. No differences in the potassium content were found between the three Gs, while with 0 and 29.1 mg K L^−1^, the potassium content in the microgreens was 53% lower compared with 58.4 mg K L^−1^ in the NS (Table 2). No difference was observed in terms of the magnesium (30 mg 100 g^−1^ FW, on average) and calcium (100 mg 100 g^−1^ FW, on average) content.

### 3.3. Proximate Analysis and Antioxidant Activity

**Figure 4:**
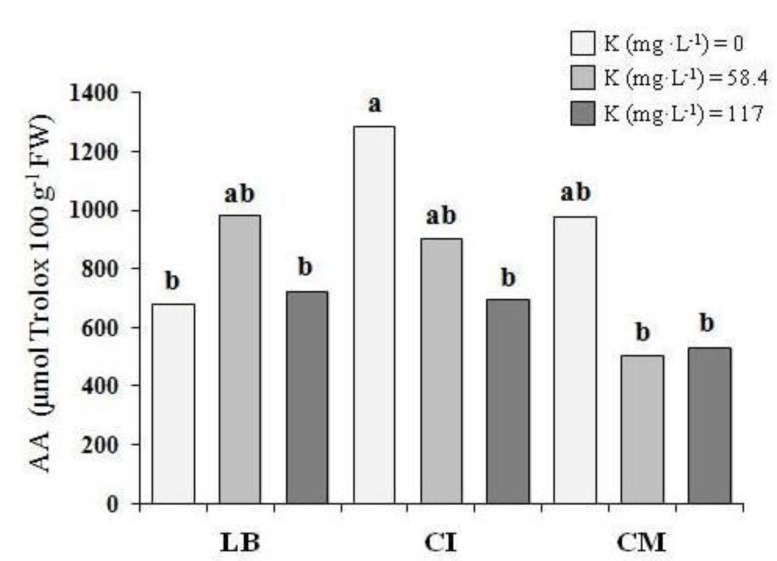
Antioxidant activity (AA) of the three genotypes of microgreens that were grown with thethree potassium levels (first experiment). LB—lettuce ‘Bionda da taglio’; CI—chicory ‘Italico a foglia rossa’; CM—chicory ‘Molfetta’. The same lowercase letters indicate that the mean values are not significantly different (*p* = 0.05).

In the first experiment, no differences were observed in terms of the total lipid (0.37 g 100 g^−1^ FW, on average) and protein (1.93 g 100 g^−1^ FW, on average) (Table 3). The K level in the NS did not affect the antioxidant activity in the LB and CM, while for the CI microgreens that were grown with 0 mg K L^−1^, an antioxidant activity value of 85% higher was shown, compared with the microgreens that were grown with 117 mg K L^−1^ (Figure 4). 

With regards to the total carbohydrate, the CI showed a content that was 25% higher when compared with the other genotypes, while the LB showed the lowest fiber content. The ashes were higher in CI compared with the LB, while no differences were observed in comparison with the CM (Table 3).

In the second experiment, no difference was observed in terms of the total lipid, protein, total carbohydrate, and ashes, with average values of 0.29, 1.79, 3.00, and 0.77 g 100 g^−1^ FW, respectively (Table 4). The microgreens of the CI showed a fiber content that was 25% higher and an antioxidant activity that was 66% higher when compared with the other genotypes (Table 4).

## 4. Discussion

In this study, for the first time, some chicory and lettuce genotypes were grown using a hydroponic system that was used for producing microgreens with low K content. We used an NS with ion concentrations that were similar to those that were reported by Hoagland [26], but at half strength. Moreover, for the potassium we also reduced the concentration until 0 mg L^−1^ was reached. We found that, independently of the genotype, in both of the experiments, the K content in the microgreens was successfully reduced by about 50% using an NS without potassium, with respect to the NS with a K concentration ≥58.4 mg L^−1^ (Table 1 and Table 2). In agreement with Di Gioia et al. [20], it was therefore possible to confirm that the mineral content in microgreens could be strongly determined by the availability of the same minerals in the provided solution. No difference was observed in terms of the shoot height, dry matter, and visual quality (Figure 2). At the same time, by reducing the potassium in the NS, we observed a lower yield, probably as a result of the fact that a potential K deficiency in plant tissues could negatively affect the stomatal opening, thereby impairing CO_2_ fixation [27]. Furthermore, when faced with this weakness, it was important to highlight the supply of K by a serving size of microgreens as well as to evaluate the K intake for the patients that were affected by chronic kidney disease. In the case of microgreens that were grown without K or with 29.1 mg K L^−1^, a serving size of 100 g supplied between 103 and 130 mg of K (Table 1 and Table 2), whereas 100 g of microgreens that were grown using an NS with a K concentration ≥58.4 mg L^−1^ supply between 225 and 250 mg of K (Table 1 and Table 2). Considering that, for the healthy population, the Institute of Medicine of the National Academies [28] had set an intake of limit of 4700 mg K per day, for the patients that were affected by chronic kidney disease, the intake from food was restricted to 1500 mg per day [7]. It was found that 100 g of microgreens, grown either without K or with low levels of K, would provide about 7.7–8.6% of the K daily intake that was recommended for the patients that were affected by chronic kidney disease, while 100 g of the microgreens that were grown using an NS with a K concentration ≥58.4 mg L^−1^ would provide about 15.8–16.5% of the K daily intake that was recommended for the patients that were affected by chronic kidney disease. Pinto et al. [29] showed a K content of 365 mg 100 g^−1^ FW for microgreens lettuces. Xiao et al. [30] indicated an average K content of 268 mg 100 g^−1^ FW for the microgreens that were obtained by several genotypes of *Brassica oleracea* L. For the arugula microgreens (*Eruca vesicaria* [L.] Cav.), some of the authors [20,30] indicated a potassium content between 301 and 343 mg 100 g^−1^ FW. While for the microgreens of basil (*Ocimum basilicum* L.) and pea (*Pisum sativum* L.), Di Gioia et al. [20] indicated a K content of 294 and 436 mg 100 g^−1^ FW, respectively. To the best of our knowledge, the literature lacked information regarding the K content in microgreens of the chicory genotypes. In addition, the results of the present study suggested a significant reduction of K in the microgreens that were destined to the patients with impaired kidney function, by reducing the K concentration in a Hoagland-like NS. It could be also interesting to compare the K content between these microgreens and the same species that were harvested at the regular stage (mature plants). Pinto et al. [29] indicated a K content of 436 mg 100 g^−1^ FW for mature lettuces, while, based on the average data that were reported from the National Nutrient Database of the United States Department of Agriculture [31], 100 g of mature leafy chicory supplied about 420 mg 100 g^−1^ FW. Thus, it was possible to highlight the very high positive impact of the reduced potassium concentrations in a nutrient solution, in terms of the microgreens production with a low K content for the patients with impaired kidney function. Moreover, according to the EC Regulation 1924/2006 on nutrition and health claims made about foods [32], the micro chicory and micro lettuce that were obtained in this study could also have been labeled with the nutritional claim ‘reduced potassium’. This was because the reduction in their K content was more than 30%, compared with the average K content of the same species that were harvested as mature plants. The availability of microgreens with a low potassium content could be exploited for two different objectives, namely: (i) to reduce the potassium intake for the same vegetable serving and (ii) to increase the amount of servings, without excessively increasing the potassium intake. The second objective could have been pursued since the potassium salts in fruits and vegetables contributed to the alkali-producing effects of these foods, also considering that this property was shown, in many recent studies, to have been of benefit in chronic kidney disease patients [8].

When the potassium content was drastically reduced from 117 to 0 mg K L^−1^, the average content of the sodium, magnesium, and calcium slightly increased in the microgreens (Table 1). According to some authors [33,34], the increase of these cations could have been a plant’s response by alleviating the negative effects of the potassium deficiency in the vegetable tissues. This considered the similar roles in the osmotic adjustment, as well as in the cellular pH and enzyme activation for both the potassium and magnesium cations [35]. At the same time, it should have also considered the role of sodium in replacing the potassium in both the biochemical and physiological non-specific functions [36]. It was interesting to highlight that, only for the CI in the first experiment, there was a significant sodium increase in microgreens that was observed, using an NS without potassium (Figure 3). This suggested a possible plant mechanism of Na^+^ exclusion, which depended on the genotype. Furthermore, from a nutritional point of view, the results of the present study showed that 100 g of the CI that was grown using an NS without K, supplied about 150 mg of sodium. This amount, which represented about 10% of the daily intake (1.5 g of sodium per day), was recommended by the European Food Safety Authority [4] and could be considered acceptable. In the second experiment, no difference in the sodium, magnesium, and calcium content in the vegetable tissues were observed when varying the K content in the NS, and also in the case of microgreens that were grown without potassium (Table 2). Therefore, with the exception of the chicory that was cultivated without K in the first experiment, the average sodium content in the microgreens (Figure 3 and Table 2) was similar, compared to the conventional chicory and lettuce [31]. It was possible that the different climate conditions during the second experiments, especially the higher relative humidity and lower mean temperatures during the last three days before harvesting (Figure 1), would have positively affected the growth and the same mechanisms of osmotic adjustments that had occurred during the first experiment were probably not necessary. Actually, in the second experiment, we found a generally higher yield in comparison with the first experiment at the same level of potassium in the NS (Table 1 and Table 2). Therefore, we could hypothesize that with the different climate conditions during the second experiment, the other solutes, such as sugars and amino acids, could have contributed to the osmotic adjustments [37].

Regarding the proximate composition in both of the experiments, the K level in the NS did not affect the content of the proteins, lipids, total carbohydrates, and fibers. These results suggested that by using an NS without K or with low potassium concentrations, it was possible to obtain a useful reduction of K in the microgreens, without negatively affecting an important aspect of the vegetables’ nutritional quality, such as the proximate composition. To the best of knowledge, the proximate analysis on the microgreens was carried out for the first time in the present study. Thus, from a nutritional point of view, it could be interesting to compare the proximate composition between the microgreens that were studied in this study, with the same species that were harvested at the regular stage (mature plants). Based on the average data that were reported from the National Nutrient Database of the United States Department of Agriculture [31], 100 g of chicory greens supplied about 1.7 g of protein, 0.3 g of lipids, 4.7 g of total carbohydrates, and 4.0 g of fibers. The same serving size of green leafy lettuce supplied about 1.36 g of proteins, 0.15 g of lipids, 2.87 g of total carbohydrates, and 1.3 g of fibers [31]. For both of the genotypes of chicory that were grown in this study, the content of the protein and lipid in the microgreens (Table 3 and Table 4) seemed to be similar in comparison with the mature plants of the same species. At the same time, it was possible to observe a slightly lower content of total carbohydrates and a much lower content of dietary fiber (Table 3 and Table 4). For lettuce, the content of the proteins, lipids, and total carbohydrates in the microgreens resulted in being similar in comparison with the mature plants of the same species, while the fiber content results were lower (Table 3 and Table 4). These results suggested the possibility of introducing microgreens into the diet of the patients with impaired kidney function that were limiting K intake and, at the same time, without modifying the intake of the macronutrients, in comparison with the mature plants. Moreover, considering their lower fiber content, the microgreens that were obtained in the present study could have also been proposed in the case of the patients that were affected by some gastrointestinal disorders, such as bowel colon syndrome.

With regards the antioxidant activity, generally higher levels were observed in the first experiment. It was well known that the antioxidant activity in vegetables was as a result of the presence of several phytochemicals. In this context, it was important to underline that light conditions could affect the accumulation of phytochemicals, especially in controlled growth environments [38]. In the soilless production of rocket that was grown under different types of greenhouses, Buttaro et al. [39] reported a lower antioxidant capacity in the plants that were grown with a lower light intensity. Some of the authors [40,41] showed an increase in the antioxidant compounds of microgreens when a supplemental light source was applied for three days before the harvest. In our research, during the last three days of the second experiment, we observed a lower photosynthetically active radiation (PAR) inside the greenhouse, in comparison with the first experiment (Figure 1). Therefore, we could hypothesize that this different light condition in the second experiment might have negatively affected the average antioxidant activity of the microgreens in comparison with the first experiment. In both of the experiments, the CI microgreens generally showed the strongest antioxidant activity (Table 3 and Table 4). The observed levels were comparable to those that were reported for the radish microgreens [42]. Generally, the K level in the NS did not affect the antioxidant activity, with the exception of the CI in the first experiment. Actually, when the potassium content in the NS was drastically reduced from 117 to 0 mg K L^−1^, the antioxidant activity for the microgreens of this genotype increased (Figure 4). In both of the experiments, it was possible to observe a different antioxidant activity, depending on the genotypes (Table 3 and Table 4). In a study that was aimed to evaluate how the potassium content in the NS affected the antioxidant properties in basil (*Ocimum basilicum* L.), an increase in the antioxidant activity was observed when the K increased from 1.0 to 2.0 mM K, but lower values were observed at 5.0 mM K [43]. Moreover, these authors showed that the antioxidant capacity was differently affected by potassium treatment among the three different cultivars that were studied. In agreement with these authors, the results of the present study suggested that the changes of the K level in the NS might have significantly affected the antioxidant activity of the microgreens, depending on the genotypes and light conditions.

Finally, from a commercial point of view, it was interesting to highlight the following: (i) currently, microgreens could be considered a successful product in the food market; (ii) the soilless cultivation method that was proposed in this study was very similar to that of the commercial production of microgreens and conventional vegetables; (iii) no additional cost were required for the microgreens production with low potassium content, with respect to the conventional ones.

## 5. Conclusions

Independent of the genotype, we found a useful reduction of potassium in microgreens by reducing the potassium concentration in the nutrient solution, without negatively affecting the proximate composition. Thanks to their distinctive peculiarities, the microgreens that were produced in this study could be labeled with the nutritional claim ‘reduced potassium’, without the additional costs with respect to the conventional microgreens’ production. The availability of the microgreens with a low potassium content could be exploited so as to reduce the potassium intake for the same vegetable serving or to increase the amount of the vegetable serving without excessively increasing the potassium intake for the patients with impaired kidney function. Future research activities could be aimed toward the application of this cultivation method on several other genotypes, in order to obtain a large variety of these special food products. Possible next goals may also be directed towards the assessment of the nutritional and functional changes in the microgreens components during the storage of fresh-cut products with a low potassium content. Other research activities could be aimed toward the assessment of the nutrient compounds’ bioaccessibility in microgreens with a low potassium content, using in vitro gastro-intestinal digestion. Finally, animal experiments using chronic renal disease models and/or clinical studies could be carried out for assessing the potential benefits of microgreens with a low potassium content in comparison to the conventional ones for the patients with an impaired kidney function.

## Figures and Tables

**Table 1:** Effects of genotype and potassium on yield, shoot height, dry matter, and inorganic cations of microgreens (first experiment).

	Yield	Shoot Height	Dry Matter	Na^+^	K^+^	Mg^2+^	Ca^2+^
	kg FW m^−2^	cm	g 100 g^−1^ FW	mg 100 g^−1^ FW			
**Genotype (G)**							
LB	1.49 b	3.9	6.33	20.8 b	194.4	30.7	99.2 b
CI	1.44 b	4.3	7.93	42.8 a	224.5	30.8	127.9 a
CM	1.73 a	4.0	6.88	44.5 a	195.4	25.6	95.5 b
**K** (mg L^−1^)							
0	1.15 b	3.4	8.28	61.5 a	128.6 b	38.0 a	126.8 a
58.4	1.81 a	4.4	6.88	27.0 b	224.9 a	29.8 b	111.4 ab
117	1.77 a	4.5	5.99	21.6 b	250.2 a	20.1 c	84.3 b
*Significance*							
G	*	NS	NS	***	NS	NS	*
K	*	NS	NS	*	**	**	*
G*K	*	NS	NS	*	NS	NS	NS

LB—lettuce ‘Bionda da taglio’; CI—chicory ‘Italico a costa rossa’; CM—chicory ‘Molfetta’. Within the same main effect and for each parameter, the same lowercase letters in the same column indicate that the mean values are not significantly different (*p* = 0.05). G × K significant interactions are reported in Figure 3. Significance: ***, **, and * for *p* ≤ 0.001, *p* ≤ 0.01, and *p* ≤ 0.05, respectively; NS—not significant.

**Table 2:** Effects of genotype and potassium on yield, shoot height, dry matter and inorganic cations of microgreens (second experiment).

	Yield	Shoot Height	Dry Matter	Na^+^	K^+^	Mg^2+^	Ca^2+^
	kg FW m^−2^	cm	g 100 g^−1^ FW	mg 100 g^−1^ FW			
**Genotype (G)**							
LB	1.83 c	4.6 b	5.85	13.5 b	154.4	31.6	101.1
CI	2.27 b	5.1 a	6.24	31.1 a	168.3	29.7	110.4
CM	2.53 a	5.0 a	5.29	32.1 a	157.4	28.7	88.1
**K** (mg L^−1^)							
0	2.08 b	4.5	6.01	32.5	103.2 b	33.2	107.3
29.1	2.04 b	4.9	5.06	20.6	129.7 b	26.2	90.4
58.4	2.52 a	5.3	6.30	23.7	247.1 a	30.6	101.8
*Significance*							
G	***	**	NS	***	NS	NS	NS
K	*	NS	NS	NS	*	NS	NS
G*K	NS	NS	NS	NS	NS	NS	NS

LB—lettuce ‘Bionda da taglio’; CI—chicory ‘Italico a costa rossa’; CM—chicory ‘Molfetta’. Within the same main effect and for each parameter, the same lowercase letters in the same column indicate that the mean values are not significantly different (*p* = 0.05). Significance: ***, **, and * for *p* ≤ 0.001, *p* ≤0.01, and *p* ≤ 0.05, respectively; NS—not significant.

**Table 3:** Effects of genotype and potassium on total lipid, protein, total carbohydrate, fiber, ashes, and antioxidant activity of microgreens (first experiment).

	Total Lipid	Protein	Total Carbohydrate	Fiber, Total Dietary	Ashes	Trolox
	g 100 g^−1^ FW					μmol 100 g^−1^ FW
**Genotype (G)**						
LB	0.40	1.75	3.47 b	0.49 b	0.81 b	793.2 ab
CI	0.34	2.11	4.55 a	0.79 a	1.04 a	959.4 a
CM	0.36	1.93	3.79 b	0.80 a	0.88 ab	668.5 b
**K** (mg L^−1^)						
0	0.42	2.17	4.86	0.82	0.88	978.9
58.4	0.37	1.93	3.74	0.65	0.95	794.9
117	0.32	1.68	3.20	0.60	0.90	647.3
*Significance*						
G	NS	NS	*	***	*	*
K	NS	NS	NS	NS	NS	NS
G*K	NS	NS	NS	NS	NS	*

LB—lettuce ‘Bionda da taglio’; CI—chicory ‘Italico a costa rossa’; CM—chicory ‘Molfetta’. Within the same main effect and for each parameter, the same lowercase letters in the same column indicate that the mean values are not significantly different (*p* = 0.05). A G × K significant interaction is reported in Figure 4. Significance: *** and * are for *p* ≤ 0.001 and *p* ≤ 0.05, respectively; NS—not significant.

**Table 4:** Effects of genotype and potassium on total lipid, protein, total carbohydrate, fiber, ashes, and antioxidant activity of microgreens (second experiment).

	Total Lipid	Protein	Total Carbohydrate	Fiber, Total Dietary	Ashes	Trolox
	g 100 g^−1^ FW					μmol 100 g^−1^ FW
**Genotype (G)**						
LB	0.32	1.87	3.09	0.64 b	0.73	457.0 b
CI	0.26	1.89	3.26	0.80 a	0.83	663.0 a
CM	0.30	1.64	2.78	0.64 b	0.78	344.1 b
**K** (mg L^−1^)						
0	0.30	1.80	3.28	0.69	0.69	531.5
29.1	0.25	1.59	2.52	0.61	0.69	500.7
58.4	0.31	1.97	3.19	0.77	0.93	489.0
*Significance*						
G	NS	NS	NS	**	NS	**
K	NS	NS	NS	NS	NS	NS
G*K	NS	NS	NS	NS	NS	NS

LB—lettuce ‘Bionda da taglio’; CI—chicory ‘Italico a costa rossa’; CM—chicory ‘Molfetta’. Within the same main effect and for each parameter, the same lowercase letters in the same column indicate that the mean values are not significantly different (*p* = 0.05). Significance: ** for *p* ≤ 0.01; NS—not significant.
